# Native and Freeze-Dried Bacterial Nanocellulose as Fat Replacers in Low-Fat Meat Emulsions: A Comparative Study of Techno-Functional Performance

**DOI:** 10.3390/foods15060998

**Published:** 2026-03-11

**Authors:** María Laura Balquinta, Nadia Florencia Nagai, María Eugenia Golzi, Neuvis Alejandro Pino Ibañez, Lucas Marchetti, Silvina Cecilia Andrés, Gabriel Lorenzo, Rubén Domínguez-Valencia

**Affiliations:** 1 Laboratorio de Investigación en Hidrocoloides y Matrices Alimentarias Saludables (LIHMAS), Centro de Investigación y Desarrollo en Ciencia y Tecnología de Alimentos (CIDCA), CONICET, CICPBA, Facultad de Ciencias Exactas, Universidad Nacional de La Plata, 47 y 116, La Plata 1900, Buenos Aires, Argentina; balquintamlaura@gmail.com (M.L.B.); nnagai@med.unlp.edu.ar (N.F.N.); egolzi@med.unlp.edu.ar (M.E.G.); alejandropino.pino@gmail.com (N.A.P.I.); marchetti.lucas@quimica.unlp.edu.ar (L.M.); scandres@biol.unlp.edu.ar (S.C.A.); lorenzogabriel@gmail.com (G.L.); 2 Departamento de Ingeniería Química, Facultad de Ingeniería, Universidad Nacional de La Plata, La Plata 1900, Buenos Aires, Argentina; 3 Centro Tecnolóxico da Carne de Galicia, Avd. Galicia Nº 4, Parque Tecnolóxico de Galicia, San Cibrao das Viñas, 32900 Ourense, Spain

**Keywords:** microstructure, freeze-drying, nanocellulose, meat product, fat replacer, rheology

## Abstract

Bacterial nanocellulose (BNC) is gaining interest in multiple industrial applications. BNC dehydration would improve its industrialization while affecting its techno-functional properties (water binding or gelling capacity). This work analyses this aspect in a representative food system where these are fundamental properties: low-fat sausages with pre-emulsified sunflower oil. Native (n-BNC) and freeze-dried (d-BNC) bacterial nanocelluloses were studied at different concentrations. During thermal processing, all batters exhibited the typical viscoelastic transition associated with protein gelation. Formulations containing d-BNC developed a higher final elastic moduli and a broader concentration range of structural reinforcement compared to n-BNC systems. In the cooked sausages, BNC incorporation enhanced hardness, cohesiveness, and water-holding capacity, particularly at intermediate concentrations. Micrographs showed that d-BNC led to a finer and more homogeneous microarchitecture, while n-BNC aggregated in hollows of the meat protein network. Additionally, the Pickering effect of dried BNC produced meat emulsions with smaller oil droplets in agreement with the differences in lightness detected. Results suggest that freeze-dried BNC could be a convenient and effective option for the food industry due to its low weight, longer storage period, and easy handling compared to native BNC.

## 1. Introduction

Consumers’ concerns about food quality have increased in recent decades, particularly regarding the perceived healthiness of food. Meat products are no exception, as their consumption has been associated with several adverse health outcomes, including ischemic heart disease and certain types of cancer, partly due to their high saturated fat content [1]. In this context, the partial or total substitution of animal fat with oils rich in unsaturated fatty acids has been widely investigated to determine whether the applied technological strategies can produce meat products with suitable physicochemical properties and acceptable sensory characteristics [2].

Nevertheless, if consumers accept these products, a potential market for healthier meat options could be developed [3].

Sausages are complex meat emulsions composed of two or more immiscible phases. They constitute a three-dimensional (3D) network formed by the interactions of proteins, fat, water, salts, and other components [4]. In recent years, various strategies have been developed to reduce the saturated fat content in these products while maintaining their sensory and technological properties. These approaches include the direct substitution of animal fat with vegetable or fish oils, the use of oil-in-water emulsions pre-stabilized with proteins or polysaccharides, and the incorporation of hydrocolloids as fat mimetics [5]. Among these, the total or partial replacement of solid fat with oils rich in unsaturated fatty acids, often as pre-emulsified systems, has emerged as one of the most promising strategies [6]. In addition, hydrocolloids are frequently included to mimic the textural attributes of the replaced fat and to produce stable meat emulsions [7].

The use of a low-sodium meat emulsion matrix represents an additional technological challenge, as sodium chloride plays essential techno-functional roles in these systems. NaCl is critical for the solubilization of myofibrillar proteins, which are responsible for emulsification, water and fat retention, and gelation properties that determine the final product quality [8]. Therefore, reducing the sodium content without compromising these functional attributes requires careful formulation strategies to maintain the structural integrity and stability of the meat emulsion

Cellulose is biocompatible, enhances mechanical properties, and has the ability to interact with protein in emulsion stabilization [9]. Particularly, bacterial nanocellulose (BNC), a relatively new biopolymer, is gaining keen interest in multiple industrial applications. *Komagataeibacter xylinus*, a Gram-negative strain of acetic acid bacteria, is the most commonly used due to its specific phenotype that allows for high efficiency in cellulose [10]. It produces BNC as a thin and well-ordered sheet structure of micro-fibrils with rectangular cross-sections (3–10 nm thick, 30–100 nm width, and 1–9 μm length) [11]. Although BNC is chemically identical to plant-derived cellulose, it is easier and cleaner to produce with higher purity [12]. The Food and Drug Administration (FDA) and the European Union (EU) recognize BNC as a GRAS additive, so it has considerable potential for use in the food industry. However, its food applications require the processing of BNC sheets to obtain a suspension or powder more suitable to combine with different ingredients and improve the structure of the final matrix. Processing could solve other technological problems, such as the excessive amount of water that BNC sheets contain (more than 95 g of water/100 g), which makes them complicated to commercialize and store [13]. Different drying methods to obtain a BNC with less water were studied [14]. Most of the drying processes produced alterations to the nano- and micro-structure of the fibrous material. As drying proceeds, nanocellulose fibres are no longer free to move and therefore tend to form agglomerates through intermolecular hydrogen bonds, changing their different technological properties [15]. Increments of the strands’ tensile stress, structure aggregation, water retention capacity reduction, and nanocellulose fibre network densifying constitute some of the reported modifications after nanocellulose drying [13,16].

Although bacterial nanocellulose has been incorporated into different meat systems and several drying methods have been studied, it remains unclear how post-synthetic processing—particularly freeze-drying—modifies its functional performance when incorporated into complex food matrices. Specifically, the impact of the structural state of BNC on water distribution, protein interactions, emulsion stability, and final product texture has not been systematically evaluated under equivalent cellulose concentrations. Therefore, understanding whether freeze-dried BNC behaves similarly to or differently from native BNC in meat emulsions represents a relevant technological and scientific gap.

This work aimed to comparatively evaluate native and freeze-dried BNC within the same low-fat meat emulsion system to elucidate how the structural state of BNC modulates its techno-functional performance. Specifically, we evaluated (I) the rheological behaviour of raw batters and (II) the physicochemical, textural, and microstructural properties of the resulting sausages.

## 2. Materials and Methods

### 2.1. Materials

Beef meat (top round cut that includes *Adductor femoris* and *Semimembranosus muscles*) was purchased at a local market. Two independent lots (6–7 kg from at least three different carcasses) were employed to produce low-fat and low-sodium sausages with different BNC states and levels. Visible fat and connective tissues were removed, and afterward, the meat was ground with a 0.95 cm plate (Meifa 32, Buenos Aires, Argentina). Then, it was homogenized and divided into lots (500 g each), vacuum packed in Cryovac BB4L bags (Sealed Air Co., Buenos Aires, Argentina), frozen, and stored at −20 °C until used (less than 21 days). High-oleic sunflower oil (HOSO; Granix S.A., Buenos Aires, Argentina) was used as the lipid source. All reagents and components were food-grade.

### 2.2. Bacterial Nanocellulose Production

Bacterial nanocellulose (BNC) was kindly provided by Instituto de Tecnología en Polímeros y Nanotecnología (ITPN-UBA-CONICET, Buenos Aires, Argentina) in the shape of pellicles, as produced by *Komagataeibacter xylinus* NRRL B-42, with the protocol of Foresti et al. [17]. BNC was produced in several independent batches under identical fermentation conditions. To minimize potential batch-to-batch variability, the obtained pellicles were pooled prior to further processing. The pooled material was then ground in a high-speed blender (TB76, TurboBlender, Buenos Aires, Argentina) for 10 min at 35,000 rpm and processed as a single batch. Subsequently, the resulting slurry (2.137 g dry BNC/100 g hydrated pellicles) was divided into two portions: one was used as native BNC (n-BNC), while the other was subjected to freeze-drying to obtain d-BNC (L-A-B4-C, Rificor, Buenos Aires, Argentina), according to the protocol described in Balquinta et al. [13]. In this way, both BNC states originated from the same pooled material, ensuring comparability between treatments.

### 2.3. Experimental Design and Sausage Production

A low-fat, low-sodium sausage base formulation was employed [18]. A bifactorial design was used to analyze the effect of the BNC state (n- or d-BNC) and the level (0.134, 0.267, 0.401, and 0.534 g dry BNC/100 g raw batter) on the sausage quality (Table 1). The selected n-BNC levels were defined considering the maximum allowable water addition in the formulation (25 g/100 g batter) previously reported for this type of system [18]. Therefore, the highest level (25 g of pellicle/100 g batter) corresponded to this technological limit, and the remaining levels were established as proportional fractions to generate a dose–response gradient while maintaining water balance. Freeze-dried BNC was incorporated at equivalent dry cellulose contents (0.134–0.534 g/100 g batter) to allow for direct comparison between BNC states. Additionally, a control formulation without BNC was evaluated (BNC0). The ingredients, which were kept constant for all the formulations, expressed as g/100 g of raw batter, were as follows: meat (68.07), HOSO (5.00), NaCl (0.608), KCl (0.492), sodium tripolyphosphate (TPP, 0.500), sodium erythorbate (0.045), NaNO_2_ (0.015), monosodium glutamate (0.020), ground pepper (0.200), nutmeg (0.050), and carminic acid (0.0032). The water incorporated with the n-BNC was subtracted from the tap water to add. The sausage formulation codes are given in Table 1.

Sausage production was according to the protocol described by Marchetti et al. [18]. Firstly, each frozen meat lot was thawed (24 h at 4 °C) and ground with NaCl, KCl, and TPP using a food processor (Universo, Rowenta, Erbach, Germany). The rest of the components were dissolved in cold water, the BNC (n- or d-) was included, and then homogenized with HOSO for 2 min to form an emulsion. Afterward, the emulsion was processed with the meat for 5 min. Raw batters (final T < 15 °C) were stuffed (vertical piston stuffer, Santini s.n.c., Marostica, Italy) into cellulose casing (d = 22 mm, Farmesa, Buenos Aires, Argentina), hand-linked, and placed in “cook-in” bags (Cryovac CN510, Sealed Air Co., Buenos Aires, Argentina). Links were cooked in a water bath (T = 80 °C, t = 11.5 min), cooled in an ice-water bath, and stored at 4 °C until further analysis.

### 2.4. Rheological Properties

Rheological measurements were performed with a controlled stress rheometer AR-G2 (TA instruments; New Castle, DE, USA) equipped with a Peltier heating and cooling system. The geometry of parallel plates (35 mm diameter, 1 mm gap) was selected. Raw batters were allowed to rest for 3 min at 25 °C for equilibration before starting the corresponding measurement. Batters’ exposed perimeters were covered with silicone oil to avoid dehydration, and the probe was enclosed with a chamber to prevent evaporation. Firstly, the linear viscoelasticity range (LVR) was assured by oscillatory stress sweep experiments at a fixed frequency of 1 Hz (6.28 rad/s) and three temperatures: 25 °C, 50 °C, and 75 °C. Thus, temperature and frequency sweeps were carried out within LVR.

#### 2.4.1. Thermo-Rheological Assays

Thermal sweeps were performed at a fixed frequency (6.28 rad/s) and stress (5.0 Pa) to ensure that samples remained in the LVR during all of the experiments. Samples were heated from 20 °C to 75 °C (heating rate 4.8 °C/min) and kept at 75 °C for 5 min to complete the cooking of the raw batter (sol/gel transition). Then, samples were cooled from 75 °C to 20 °C at 10 °C/min. The presented thermo-rheograms correspond to average data of at least two replicates per formulation.

#### 2.4.2. Frequency Sweeps

To accomplish frequency sweeps, raw batters were placed on the Peltier and treated as described in Section 2.4.1. Afterward, an isothermal step at 20 °C was applied for 5 min. Frequency sweeps at 20 °C of thermally treated samples were performed.

### 2.5. Physiochemical Characterization of the Sausages

#### 2.5.1. Process Yield

Yields were calculated as the weight of one link after the thermal treatment divided by the weight of the uncooked product and expressed as g/100 g (8 replicates per formulation were tested).

#### 2.5.2. Water Activity and Water-Holding Capacity

The water activity (aw) of cooked sausages was measured with an Aqualab 4TEV (Decagon Devices Inc., Washington, DC, USA), at least in triplicate. Water-holding capacity (WHC) was measured as liquid released after centrifugation [18].

#### 2.5.3. Colour

The colour of recently transversally cut sausages’ surfaces was measured using a ChromaMeter CR-400 (Minolta Co., Ramsey, NJ, USA) at room temperature. CIE-LAB parameters (lightness, L* redness, a*; and yellowness, b*) were determined by measuring 12 replicates per formulation. Additionally, ΔE was obtained as $\Delta E=\sqrt{{\left(\Delta L^{*}\right)}^{2}+{\left(\Delta a^{*}\right)}^{2}+{\left(\Delta b^{*}\right)}^{2}}$.

#### 2.5.4. Texture Profile Analysis

Texture Profile Analysis (TPA) of sausages was performed with a TAXT2i Texture Analyzer (Stable Micro Systems, Surrey, UK) and a 75 mm diameter probe at 0.5 mm/s (SMSP/75) at 20 °C. At least eight replicates from each sample (1.5 cm thick and 1.7 cm diameter) were cut from the centre of the links and compressed to 30% of their original height in two consecutive cycles. From the force–time curve, hardness, springiness, cohesiveness, chewiness, and resilience were calculated.

### 2.6. Confocal Scanning Laser Microscopy (CSLM)

The microstructure of each sample was observed using confocal fluorescence microscopy LEICA TCS SP5 (Mannheim, Germany). Firstly, 20 μL of fat-soluble stain (Nile Red) was used for 5 h, and then a mixture of rhodamine B (0.001 g/100 g) and calcofluor white (0.01 g/100 g) was used for proteins and cellulose fibres, respectively. Samples rested for 24 h within a closed recipient (darkness), washed with distilled water, and covered with a glass cover slip. Images were acquired using a 20× HCX PL APO CS water immersion objective.

### 2.7. Statistical Analysis

**Figure 1:**
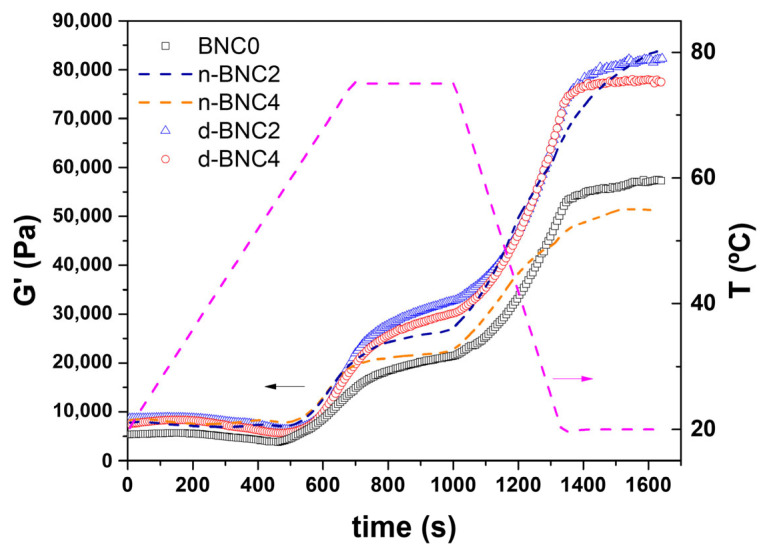
Temperature sweeps (G′ vs. time) of low-fat, low-sodium raw sausages’ batters. Formulations are coded according to Table 1.

**Figure 2:**
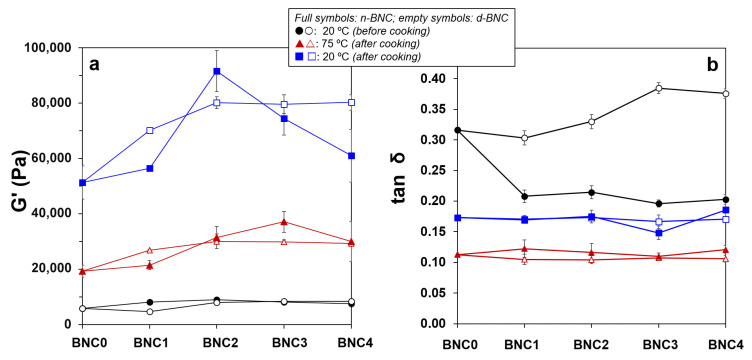
Storage moduli (**a**) and loss tangent (**b**) of meat emulsions as a function of the bacterial nanocellulose (BNC) content during thermal processing. Error bars indicate standard error of the mean.

**Figure 3:**
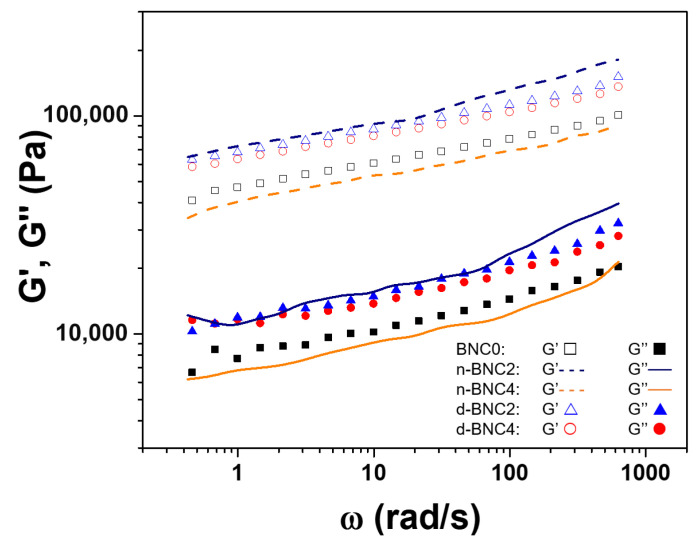
Frequency sweeps of low-fat, low-sodium meat sausages containing different concentrations and states of bacterial nanocellulose (BNC). Formulations are coded according to Table 1.

**Figure 4:**
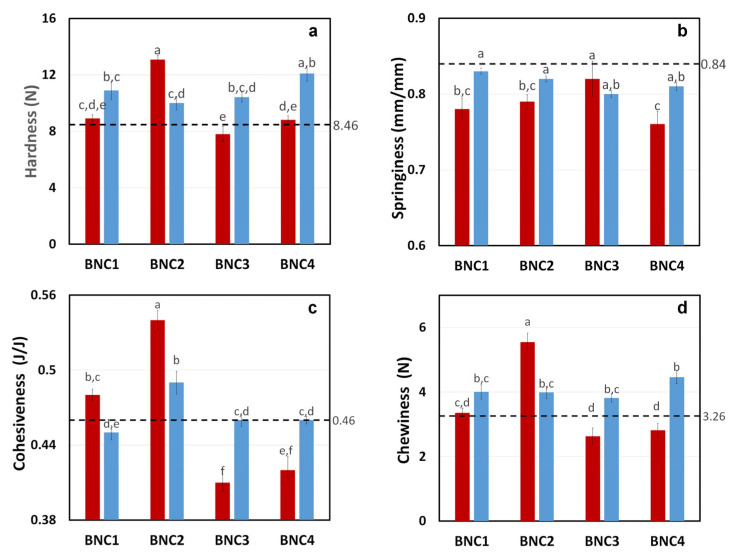
Texture parameters of low-fat, low-sodium meat sausages with different states and levels of bacterial nanocellulose: (**a**) hardness; (**b**) springiness; (**c**) cohesiveness; and (**d**) chewiness. ■ n-BNC; ■ d-BNC. Different letters on the bars correspond to significant differences among samples (*p* < 0.05). Error bars indicate standard error of the mean. Dashed lines correspond to the textural results for control formulation (BNC0).

The entire trial was performed in duplicate. Analyses of variance were conducted separately on dependent variables considering a two-way factorial design (analyzing main effects and interactions). Differences in means and F-tests were considered significant when *p* < 0.05. Tukey’s test was chosen for simultaneous pairwise comparisons. All statistical procedures were computed using InfoStat v2009 software (Infostat 2008, Infostat Group, Universidad Nacional de Córdoba, Argentina). Artwork in the present paper was created employing Origin Lab (Figure 1 and Figure 3) and Excel (Figure 2 and Figure 4).

## 3. Results and Discussion

### 3.1. Rheological Analysis

The rheological behaviour of meat batters during thermal processing provides critical insight into the structural changes that govern the final quality of emulsified sausages. Figure 1 shows the evolution of the storage modulus (G′) during a thermal sweep that simulates the cooking process for the control batter and selected formulations containing n-BNC or d-BNC.

During the initial heating stage (up to ~500 s), all samples exhibited a qualitatively similar behaviour. A slight decrease in G′ was observed until approximately 58 °C, which is associated with the denaturation of myosin tails, leading to a transient increase in matrix fluidity and a partial disruption of the protein network formed at lower temperatures [19]. This was followed by a sharp increase in G′ due to the thermal aggregation of myofibrillar proteins, marking the sol–gel transition and the formation of a three-dimensional protein network [20].

A notable effect of the BNC state became evident during the isothermal stage at 75 °C (between 700 and 1000 s in Figure 1). At equivalent cellulose levels, batters with d-BNC reached higher G′ values than those with n-BNC. This can be explained by differences in water availability. Batters containing d-BNC have more free water available, which facilitates the unfolding and subsequent cross-linking of meat proteins. Conversely, in n-BNC batters, a significant portion of the water is tightly bound within the nanocellulose pellicles, making it less available for protein hydration and network formation, thus resulting in a less elastic gel. Sun et al. [21] stated that the intertwined network formed in a nanocellulose–protein complex could enhance gelation, resulting in higher viscoelastic properties, but this effect appears to be modulated by the hydration state of the cellulose. During the subsequent cooling stage (between 1000 and 1300 s), a further sharp increase in G′ was observed, indicative of the strengthening of the permanent gel network through hydrogen bonding and further protein aggregation [19,20].

Figure 2 provides a comprehensive overview of the batters’ viscoelastic properties at three key stages of processing (raw at 20 °C, after cooking at 75 °C, and after cooling to 20 °C) as a function of BNC concentration and state. In the raw batters (20 °C before cooking), the elastic modulus (G′) remained practically invariant regardless of the nanocellulose content in the matrix (Figure 2a). However, a closer examination of the loss tangent (tan δ = G″/G′) at this stage revealed notable differences depending on the BNC state (Figure 2b). The incorporation of n-BNC resulted in more elastic batters (lower tan δ, <0.22) compared to the control (BNC0), and this effect was independent of the n-BNC concentration. In contrast, d-BNC did not significantly modify the batters’ viscoelastic character at lower levels (d-BNC1 and d-BNC2). A further increase in d-BNC content led to batters with a more viscous character (higher tan δ). This could be attributed to the limited swelling capacity of the dried BNC fibres at room temperature, which may initially disrupt the protein network’s organization without contributing to its elastic structure. Flores et al. [22] reported a similar trend in emulsified meat systems, where the addition of starch, which has a thickening rather than gelling effect before heating, did not increase G′. At the end of the cooking process (75 °C, after cooking), all sausages exhibited a well-developed gel network with a tan δ of ~0.11 (Figure 2b), confirming the dominance of elastic behaviour. After cooling to 20 °C, the G′ values were significantly higher than at 75 °C (Figure 2a), and the differences between BNC states became even more pronounced. The incorporation of d-BNC led to a concentration-dependent increase in the final G′ of the cooked sausages, tending toward a plateau at the highest incorporation levels. In contrast, n-BNC showed a distinct non-linear behaviour: a marked increase in G′ was observed only at the intermediate concentration (n-BNC2, 0.267 g/100 g), whereas the other levels (n-BNC1, n-BNC3, and n-BNC4) resulted in G′ values lower than those of the corresponding d-BNC formulations and, in some cases, similar to the control.

This differential behaviour was also evident in the mechanical spectra of the cooked products at 20 °C (Figure 3). All formulations displayed a typical solid-like spectrum, with G′ consistently exceeding G″ across the entire frequency range and showing only a slight frequency dependence (slope < 0.25), which is characteristic of a stable, elastic gel [23]. The reinforcing effect of d-BNC was clear, as the spectra shifted progressively towards higher moduli with increasing d-BNC content. This suggests that d-BNC, being finely dispersed, effectively fills the interstitial spaces within the meat protein network, leading to a more compact and rigid structure without disrupting the gel matrix [20,24]. Hu et al. [20] found similar results using regenerated cellulose in meat emulsions, observing that fat substitution resulted in a more rigid and firmer gel than the control without cellulose. On the other hand, the behaviour of n-BNC formulations confirmed the pattern observed in Figure 2a. Only n-BNC2 produced a more elastic system than the control (BNC0), while n-BNC1 and n-BNC4 resulted in mechanical properties similar to BNC0. This indicates that the ability of n-BNC to reinforce the meat matrix is confined to a very narrow concentration range. The contrasting behaviours of n-BNC and d-BNC can be mechanistically explained by a competition for available water and the physical state of the cellulose itself. BNC has been described as a molecule with a very high water-binding capacity due to its physical entanglement, aggregation, and supramolecular interactions [13]. In native BNC, water is an integral part of its structure, bound within the pellicle’s network, and it is unlikely to release a considerable amount of water to the protein phase during heating. Therefore, the higher the n-BNC level in a formulation, the lower the water available for meat protein unfolding, 3D bonding, and, consequently, the meat emulsion stabilization is affected. Conversely, freeze-dried BNC is incorporated as a dry material that must rehydrate in situ. The drying process may also result in a more compact structure with a reduced water-binding capacity compared to the native pellicle [12]. This allows for a more balanced distribution of water between the protein and the cellulose phases, enabling the meat proteins to form a robust gel, while the rehydrated d-BNC fibres, being smaller and more uniformly dispersed, integrate into the protein matrix to form a reinforced, co-continuous network across a much wider concentration range. In essence, d-BNC acts as a structural filler that strengthens the gel without starving the protein network of essential water, whereas n-BNC acts as a water competitor whose large, aggregated clusters can disrupt network homogeneity at suboptimal concentrations.

### 3.2. Physicochemical Characteristics

The process yield reflects the water and oil retention of a meat emulsion after cooking and cooling and its stability. All of the sausage formulations presented high yields with an average value of approximately 97.5 g/100 g and no significant differences among them (*p* > 0.05). Water activity did not show significant differences with neither the state of BNC nor the concentration, averaging approximately 0.98. On the other hand, the sausages presented different WHC values (Table 2). Compared to the control, the hydrocolloid incorporation increased the WHC except for n-BNC1 which did not significantly change concerning BNC0 (Table 2). Native or dried BNC2 sausages presented the highest WHC, showing a tendency to decrease with an increasing BNC concentration, although differences were not statistically significant (*p* > 0.05). The optimal WHC at intermediate BNC levels suggests a balance between hydrocolloid–water binding and protein network development. Moderate incorporation levels could enhance matrix structuring, as reflected in rheological results, whereas higher n-BNC additions could increase water competition, slightly limiting protein gel efficiency. This interpretation is consistent with the notion that water may be differently distributed between meat proteins and BNC molecules depending on the incorporation level. Similar results were reported for different emulsified meat systems stabilized with bacterial nanocellulose. Frankfurt-type sausages [25], mahi-mahi surimi [26], and Chinese-type meatballs [27] showed a WHC higher than the controls. The WHC was also increased for sausages with pre-emulsified soybean oil with 2 g/100 g of vegetable cellulose from different sources [28].

The low-fat, low-sodium sausage without BNC (BNC0, control) presented an L* value of 61.50 ± 0.21, which is similar to that reported by other authors in similar systems [29,30]. The addition of d-BNC significantly increased (*p* < 0.05) the lightness of the sausages (Table 2). This increase could be related to more water content or more evenly distributed water, leading to a smoother surface. It has also been reported that an increase in L* could be related to the smaller oil globules of pre-emulsion, which had a larger surface area to reflect light [31]. In contrast, n-BNC addition showed no significant differences with the control (even though there was a slight decrease in the value of L*). Regarding the redness and the yellowness, no marked differences were found among formulations with BNC in regard to the control (Table 2). Similar findings in these chromatic parameters were described by Qi et al. [28], where emulsion-type sausages showed no noticeable differences in colour parameters. The overall colour change in the sausages (ΔE) was controlled by the modifications in lightness mentioned above, which ended up showing that those formulated with added d-BNC were the ones that presented appreciable colour differences that could be detected by untrained consumers.

The texture parameters of different sausage formulations are presented in Figure 4. Hardness is one of the most important textural characteristics of emulsified meat products. When analyzing the effect of the BNC state on sausage hardness, adding d-BNC produced an overall increase compared to n-BNC. However, the interaction between the state and level of BNC was significant (*p* < 0.05), as can be seen in Figure 4a. Thus, d-BNC led to emulsified meat products with a similar resistance to compression regardless of the BNC level. Native BNC exhibited similar behaviour to the one observed in the viscoelastic analysis, i.e., a marked increase in hardness with the intermediate content (n-BNC2), followed by a sharp decrease with a further BNC concentration in the matrix. The control sample resulted in softer sausages (Hardness_BNC0_ = 8.46 ± 0.42 N), which implies that the hydrocolloid incorporation led to a reinforcement of the structure that could also contribute to an increase in the water-holding capacity.

In the case of springiness (Figure 4b), an overall analysis seems to show that BNC addition caused a reduction in this characteristic regarding the control (Springiness_BNC0_= 0.84 ± 0.01). However, d-BNC samples showed more elastic responses than those containing n-BNC. This less springy behaviour of the n-BNC sausages could be attributed to the less effective integration of the native BNC pellicle in the meat protein matrix. Zhou et al. [32] found less springy emulsified meat systems when carrageenan was added but a dual behaviour similar to the BNC effect described in this paper when flaxseed gum was included. Lin and Lin [27] reported hardness and springiness reductions with no changes in cohesiveness for Chinese-style meatballs when BNC was included.

When cohesiveness was analyzed for the different low-fat, low-sodium sausage formulations (Figure 4c), a significant effect of the state-level interaction of BNC was observed. Results of the sausages’ cohesiveness showed a remarkably similar trend to their rheological behaviour. Native BNC incorporation produced a significant increase in cohesiveness at low concentrations compared to the control (Cohesiveness_BNC0_ = 0.46 ± 0.01). However, higher incorporation levels (n-BNC3 and n-BNC4) resulted in a sharp decrease, indicating some sort of negative interaction or incompatibility among the meat protein gel and the native BNC under this condition (less water available). Increasing the n-BNC level beyond an optimum could result in a significant hindrance in the meat protein gel’s homogeneity, causing the cohesiveness to decrease. On the other hand, dried BNC appeared to have the same effect regarding n-BNC on the sausages’ cohesiveness at low concentrations, but higher (d-BNC3 and d-BNC4) concentrations did not show significant differences from the control. The incorporation of d-BNC fibres could be more evenly distributed into the batter, reducing the magnitude of the hydrogel cluster formation and contributing to a more homogeneous matrix without altering the cohesiveness of the meat protein gel.

The sausages’ chewiness profiles (Figure 4d) resulted in similar hardness and cohesiveness. All d-BNC levels resulted in sausages with a higher resistance to the mastication process, but only n-BNC2 could surpass the control.

Finally, no significant effects of the BNC state, level, or their interaction were observed for resilience (*p* > 0.05). All formulations exhibited similar resilience values (approximately 0.27), indicating that the immediate elastic recovery after deformation was not markedly affected by BNC incorporation regardless of its structural state. This suggests that, although BNC modified hardness and cohesiveness, it did not alter the short-term elastic response of the protein gel network.

### 3.3. Microstructure of Meat Emulsions

**Figure 5:**
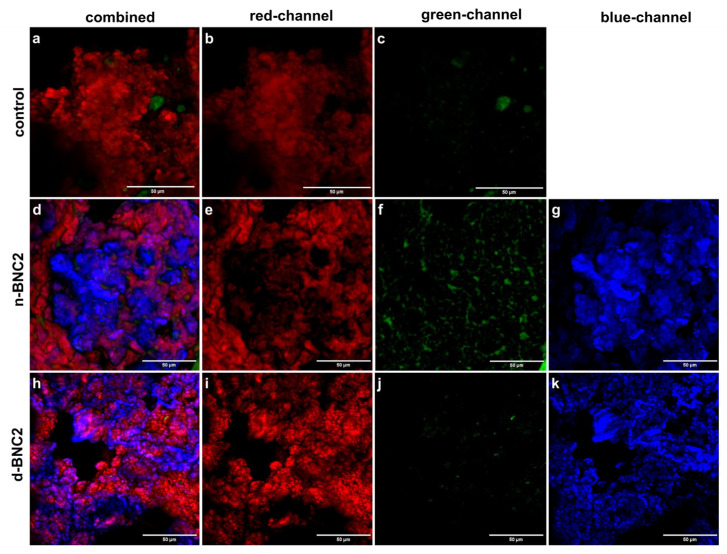
Confocal scanning laser microscopy images of low-fat meat sausages with different types of BNC: (**a**–**c**) BNC0; (**d**–**g**) d-BNC; and (**h**–**k**) n-BNC. Blue channel: calcofluor white; red channel: rhodamine B; and green channel: nile red. Scale bars = 50 µm.

Microscopic observations of different BNC-added sausages showed a typical meat emulsion microstructure (Figure 5). Particularly, control sausages exhibited a structure of a dense and homogeneous network of meat proteins, observed as the red fluorescent regions stained with Rhodamine B, with few and/or small cavities corresponding with the dark areas (Figure 5a–c). Nile red (green channel) dyed the lipid phase of the meat emulsions embedded within the meat protein gel.

The micrographs of meat emulsions containing BNC provide information about the impact of the hydrocolloid addition in the systems and could be useful to explain the differences observed in the mechanical properties when different states of BNC were included (Figure 5d–k). The intermediate level (BNC2) was selected for microstructural illustration because it showed the most pronounced differences in rheological and textural behaviour.

In general, the BNC formed a secondary network, which could be correlated with the higher hardness or viscoelastic properties of the BNC-added sausages. The cross-linked matrices, formed between the nanocellulose and the protein gel, could help to entrap more fat droplets and form a reinforced structure that leads to better emulsion stability with higher yields and WHC regarding a control formulation [33]. Micrographs of sausages with different BNC levels did not show marked differences in their structure. Nevertheless, the BNC state had a significant impact on the microarchitecture of the system. Dried BNC produced a finer and more homogeneous structure (Figure 5h–k), reflected in the extended co-localization of the blue and red channels (fuchsia regions in the combined image). It could be observed that the nanocellulose fibres, displayed around the meat protein aggregates, lead to a more evenly distributed network. It has been reported that highly dispersed nanocellulose might form a rigid network and cross-link with the protein matrix, which helps to entrap more fat droplets and form a more homogeneous structure [34].

Conversely, native BNC-added sausages presented a structure of large clusters of BNC pellicles, located in the hollows of the meat protein network (Figure 5d–g).

This type of microstructure was also described by Gibis et al. [24] for microcrystalline cellulose in fried beef patties, where the hydrocolloid filled the gaps of the tight meat fibre network. This more heterogeneous structure could explain the changes in the sausage’s cohesiveness. When an excess of n-BNC was included, the pellicles clusters could affect the structure of the system, since there would be no more space in the gaps of the meat protein network, and its entanglement could be diminished to include the excess of BNC.

This same mechanism of interaction between a hydrocolloid and the meat protein network was proposed by Zhuang et al. [35], who described the inclusion of sugarcane fibre in the cavities, which led to improved textural properties, but large amounts of fibre would disrupt the aggregation of myofibrillar proteins.

On the other hand, d-BNC particles appeared to be smaller than n-BNC; also, d-BNC was more finely and homogeneously dispersed in the matrix. This allowed the d-BNC network to be integrated with the meat protein network. Thus, the range of concentration where d-BNC improved the physicochemical characteristics was wider than n-BNC, since a higher level of hydrocolloid would be needed to disrupt the protein network.

Other important aspects of the emulsified meat products are the fat/oil droplet size and distribution. Jiménez-Colmenero et al. [33] reported that the microstructure of frankfurter with O/W emulsion stabilized with sodium caseinate had smaller cavities and a firmer network than a fat control.

When the microstructure of low-fat, low-sodium sausages was studied, it was observed that the state of BNC affected the emulsion formation, since the oil droplets in d-BNC are noticeably smaller than n-BNC (where signs of coalescence could be appreciated), related to a more stable emulsion (Figure 5f,j). Dried cellulose nanofibers were reported to produce model meat emulsions with a more compact structure and smaller oil droplets [28]. The authors attributed this behaviour to an enhancement of the interactions between meat proteins and fat caused by the Pickering effect and the structure formation of the nanofibers.

Furthermore, the observed reduction in droplet size would lead to an increase in the exposed surface area and could explain the higher lightness detected in sausages with dried bacterial nanocellulose (higher L* and ΔE).

## 4. Conclusions

Bacterial nanocellulose proved to be an effective techno-functional ingredient in low-fat, low-sodium meat emulsions, significantly influencing rheological behaviour, textural attributes, and water retention, depending on its structural state and incorporation level. Native BNC improved product structure within a narrow concentration range, whereas freeze-dried BNC exhibited a broader window of functional performance, promoting stronger gel networks, enhanced hardness and cohesiveness, an improved water-holding capacity, and a more homogeneous microstructure with smaller oil droplets.

The superior dispersion, extended concentration tolerance, reduced water content, lower weight, and improved handling characteristics of freeze-dried BNC highlight its advantages for industrial application compared to its native hydrogel form.

Although sensory perception and long-term stability were not evaluated in this study, the observed modifications in textural parameters and water retention may have implications for sensory attributes such as firmness and juiciness, which should be confirmed in future investigations.

## Figures and Tables

**Table 1:** BNC states and levels and additional water used in different sausage formulations.

Sausage Formulation Code	BNC State	n-BNC	d-BNC	BNC Content *	Water
		(g/100 g Batter)			
BNC0 (control)	-	-	-	0	25.00
n-BNC1	Native	6.25	-	0.134	18.75
n-BNC2		12.50	-	0.267	12.5
n-BNC3		18.75	-	0.401	6.25
n-BNC4		25.00	-	0.534	0
d-BNC1	Freeze-dried	-	0.134	0.134	24.87
d-BNC2		-	0.267	0.267	24.73
d-BNC3		-	0.401	0.401	24.60
d-BNC4		-	0.534	0.534	24.47

* BNC content reflects the final solid BNC concentration in the system regardless of its state.

**Table 2:** Water-holding capacity (WHC), colour parameters (L*, a*, and b*), and ΔE of low-fat, low-sodium meat sausages ^†^.

Formulation	WHC (g/100 g)	L*	a*	b*	ΔE
BNC0 ^‡^	74.24 ± 1.22	61.50 ± 0.21	13.86 ± 0.12	10.63 ± 0.07	0
n-BNC1	74.40 ± 0.41 ^b^	61.37 ± 0.23 ^a,b^	12.62 ± 0.17 ^a^	10.97 ± 0.08 ^d^	1.52 ± 0.12 ^a^
d-BNC1	80.50 ± 0.9 ^a,b^	64.03 ± 0.24 ^c^	14.12 ± 0.14 ^b^	10.00 ± 0.09 ^a^	2.69 ± 0.22 ^b^
n-BNC2	83.80 ± 0.58 ^a^	60.67 ± 0.19 ^a^	12.91 ±0.17 ^a^	11.00 ± 0.07 ^d^	1.53 ± 0.18 ^a^
d-BNC2	83.02 ± 1.36 ^a^	64.41 ± 0.29 ^c^	13.21 ± 0.16 ^a^	10.23 ± 0.06 ^a,b^	3.13 ± 0.22 ^b,c^
n-BCN3	78.67 ± 1.44 ^a,b^	61.37 ± 0.25 ^a,b^	12.83 ± 0.17 ^a^	10.83 ± 0.06 ^c,d^	1.36 ± 0.14 ^a^
d-BNC3	78.15 ± 1.16 ^a,b^	64.59 ± 0.11 ^c^	13.10 ± 0.12 ^a^	10.63 ± 0.06 ^c^	3.21 ± 0.1 ^b,c^
n-BNC4	78.89 ± 1.38 ^a,b^	61.06 ± 0.18 ^a,b^	12.55 ± 0.2 ^a^	10.79 ± 0.07 ^c,d^	1.61 ± 0.16 ^a^
d-BNC4	78.29 ± 1.18 ^a,b^	64.92 ± 0.14 ^c^	13.15 ± 0.23 ^a^	10.50 ± 0.12 ^b,c^	3.59 ± 0.17 ^c^

^†^ Different superscripts within the same column indicate significant differences among samples (*p* > 0.05). Formulation codes are in Table 1. Values are reported as mean ± standard error of the mean. ^‡^ BNC0 is shown as reference and was not included in the two-way ANOVA (BNC state × level). Superscripts indicate differences among BNC-containing formulations.

## Data Availability

The original contributions presented in the study are included in the article, further inquiries can be directed to the corresponding author.
